# Amelanotic Melanoma Invading the Heart

**DOI:** 10.7759/cureus.61714

**Published:** 2024-06-05

**Authors:** Ahmed Kazi, Abeera Akram, Sana Hyder

**Affiliations:** 1 Internal Medicine, Saint Francis Hospital, Hartford, USA; 2 Cardiology, University of Connecticut, Farmington, USA; 3 Cardiology, Hartford Hospital, Hartford, USA

**Keywords:** cardio-oncology, cardiology research, amelanotic melanoma, echocardiography in cardio-oncology, cardiac melanoma

## Abstract

We present a case of a middle-aged woman who initially presented with shortness of breath but was ultimately found to have a large mass-like lesion in the right atrium of the heart with multi-modality imaging including cardiac computed tomography, cardiac magnetic resonance imaging, and echocardiogram. Biopsy results were positive for amelanotic melanoma. The patient underwent extensive debridement surgery, and she was started on chemotherapy with a close follow-up with an oncologist. In the setting of an aggressive course of disease, unfortunately, the patient passed away secondary to sudden cardiac arrest.

Cardiac melanoma, also known as melanoma of the heart, is an extremely rare type of melanoma that originates in the heart. This case attributes to the professional growth and competency of healthcare providers involved in the care of patients with cardiac melanoma, ultimately aiming to optimize patient outcomes and quality of life. Due to its rarity and the challenges associated with its diagnosis and treatment, prognosis for cardiac melanoma is generally poor. However, advancement in cancer research and treatment may offer hope for improved outcomes in some cases.

## Introduction

Cardiac tumors are abnormal growth in the cardiac muscle or adjacent structures and are known to be great masquerades. Patients presenting with cardiovascular or constitutional symptoms have an incidental discovery of a cardiac mass after post-mortem or while imaging for any other pathology [1]. Cardiac tumors are broadly divided into benign mass and malignant mass. Cardiac myxomas are the most common benign tumor originating in the left atrium of the heart [2]. Malignant tumors can be primary or secondary. Primary cardiac tumors are very rare with a frequency of 0.001-0.003% [1]. Secondary malignant tumors are usually metastatic spread of malignant melanoma, primary breast cancer, or lung cancer.

In a retrospective cohort study by Alexander et al., they found that cardiac spread of melanoma occurs in less than 2% of the patients with metastatic melanoma. It manifests as small masses, large tumors, or infiltrative masses in the cavity. It could also involve the pericardium and epicardium of the heart [3]. As metastatic tumor cells spread through the superior or inferior vena cava, involvement of the right ventricle is expected more but as per the literature review by Alexander et al., the most common site was the left ventricle [4].

Prognosis of cardiac melanoma is very poor. The average time of death from the time of diagnosis is two years [5]. Hence, early detection and treatment of melanoma are very important when the disease is more curable.

## Case presentation

A 58-year-old female presented with dyspnea on exertion. Her medical history included stage II b malignant melanoma, for which she underwent wide excision with a negative sentinel lymph node biopsy two years ago. Additionally, she tested negative for the BRAFV600E/K mutation at that time. She also had a history of hyperlipidemia and a recent COVID-19 infection. The patient reported experiencing worsening shortness of breath for nearly one week, which significantly limited her daily activities. She also complained of fatigue. However, she denied having lower extremity edema or orthopnea. Upon physical examination, the patient was observed to be an obese woman with a blood pressure of 138/64 mmHg and a heart rate of 80 bpm. Her oxygen saturation level was 98% on room air, and she exhibited a normal respiratory pattern. Cardiac examination revealed normal, regular S1-S2 heart sounds without any murmurs detected. During the chest examination, there were no wheezes or rales noted in the bilateral lung fields. On examination of the lower extremities, there was no edema observed. Laboratory results on admission revealed mild leukocytosis and an elevated Pro-BNP level of 2040 pg/ml as shown in Table 1.

**Table 1:** Lab values on admission BNP: B-type natriuretic peptide

Lab test	Lab value	Normal range	Unit
White blood cell	12.9	4.0-11.0	Thou/uL
Hemoglobin	10.2	11.7-15.7	g/dl
Hematocrit	31.6	35.0-47.0	%
Sodium	135	136-145	mmol/L
Potassium	3.9	3.4-5.3	mmol/L
Chloride	100	98-107	mmol/L
Bicarbonate	24	22-33	mmol/L
Creatinine	0.7	0.7-1.1	mg/dl
Troponin	8	<15	ng/l
Pro-BNP	2040	<125	pg/ml

**Figure 1:**
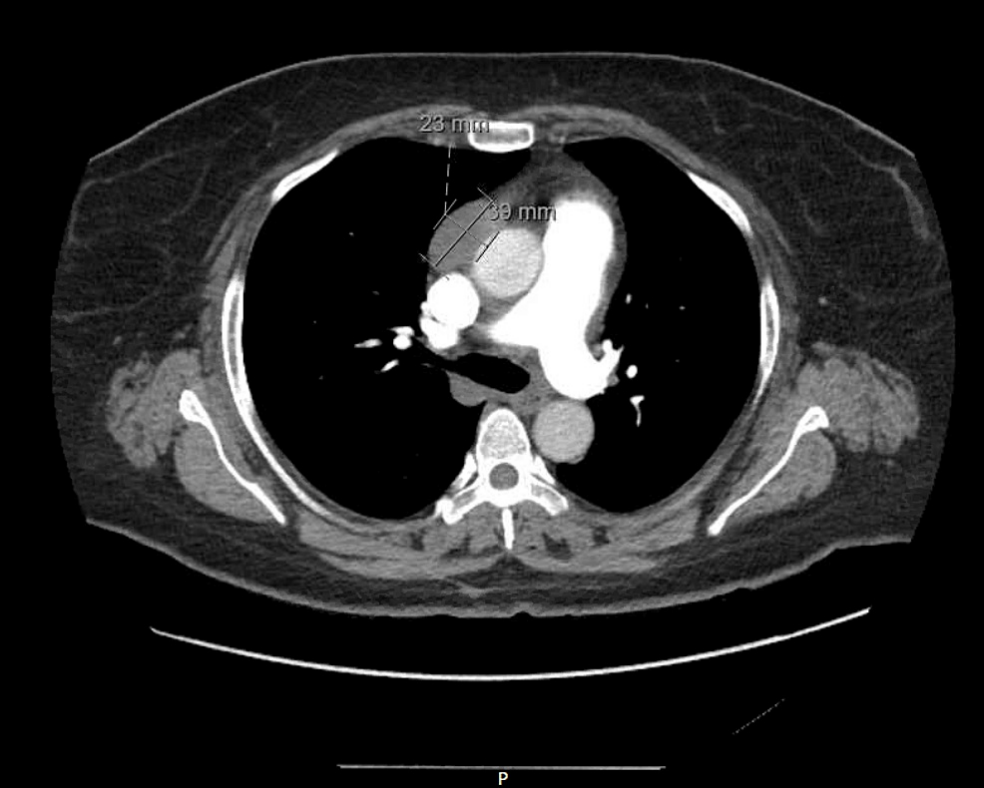
CTA showing mass in the right atrium. CTA: Computed tomography angiogram

The CT angiogram (CTA) of the chest revealed a large mass-like lesion located in the right atrium extending into the right ventricle and the inferior vena cava (IVC) (Figure 1).

**Figure 2:**
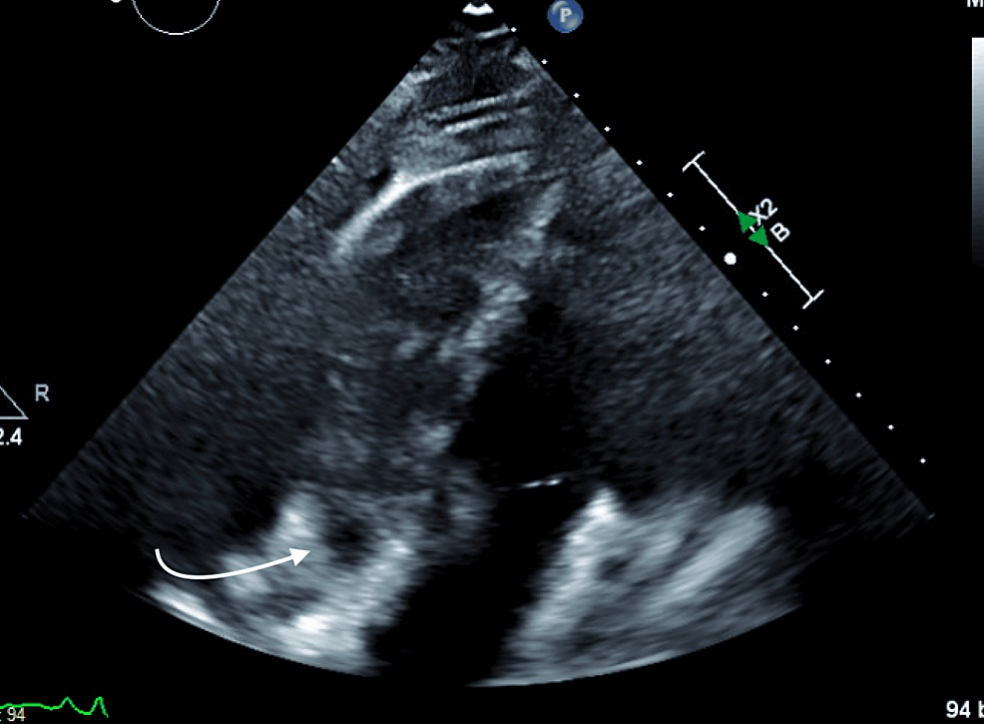
Transthoracic echocardiogram showing right atrium mass.

**Figure 3:**
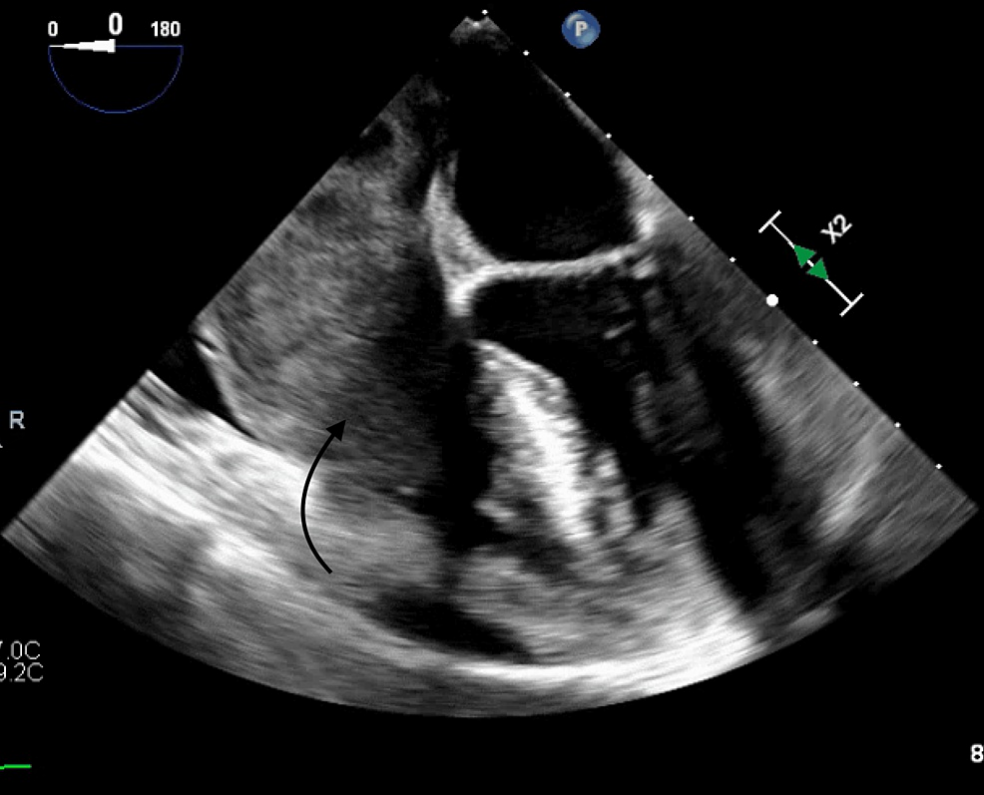
Transesophageal echocardiogram showing right atrium mass extending into the right ventricle.

**Figure 4:**
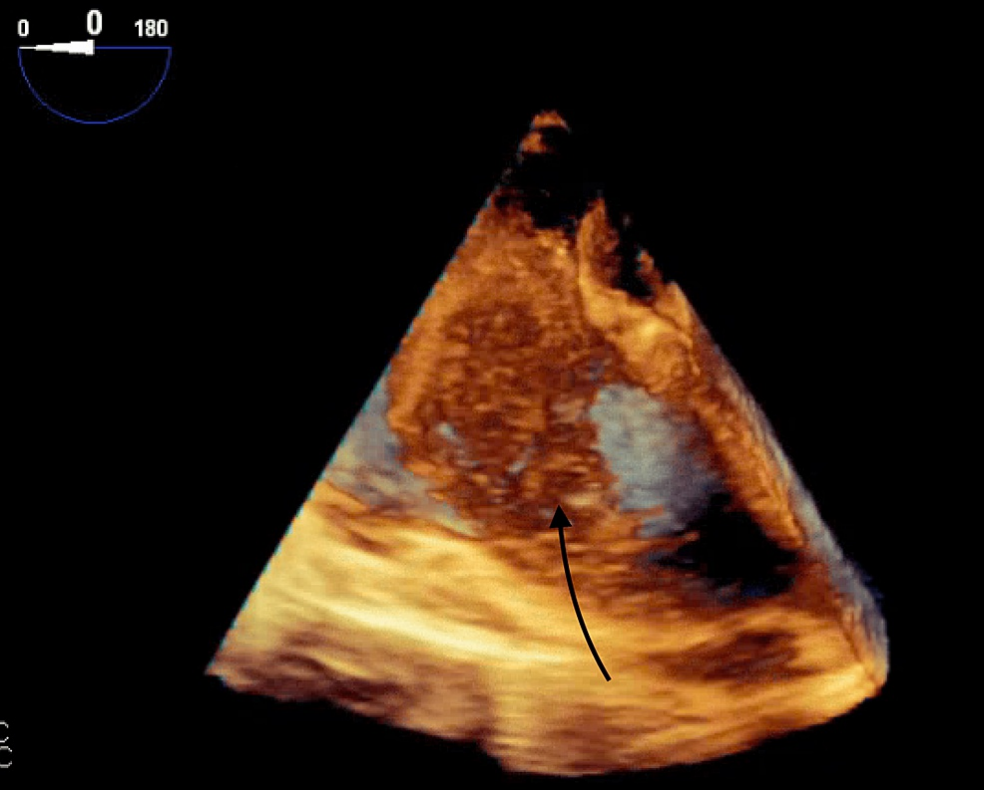
3D image of the right atrium mass.

**Figure 5:**
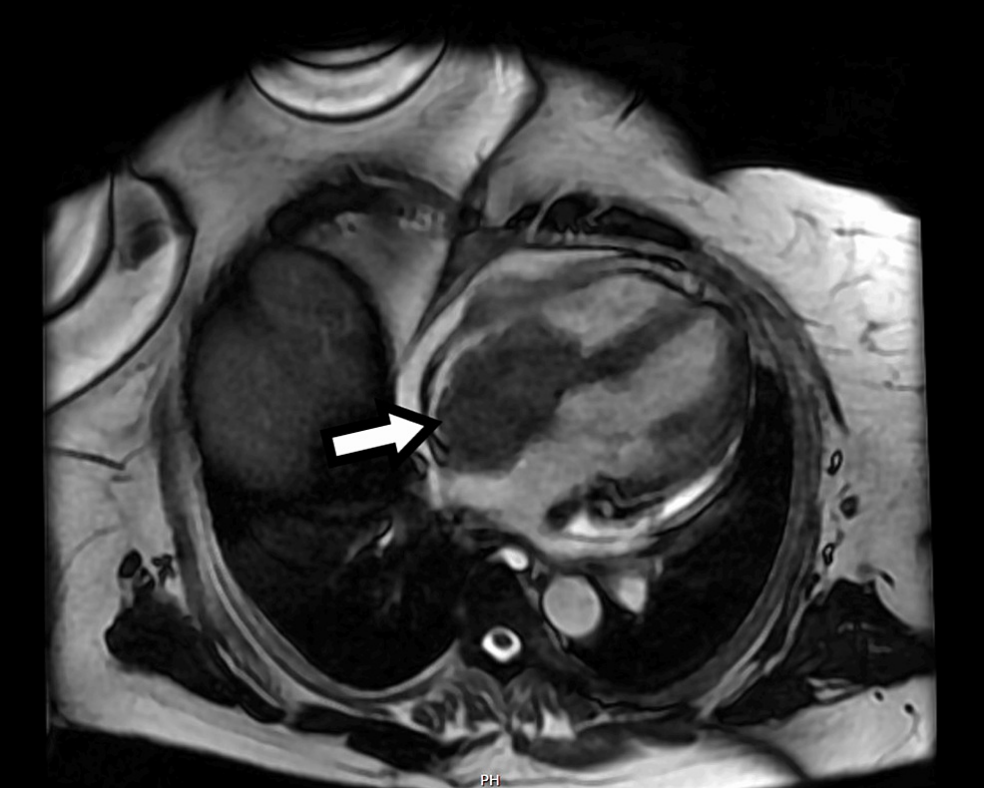
MRI showing the mass in the right atrium

Further imaging studies included a transthoracic echocardiogram (Figure 2), a transesophageal echocardiogram, a 3D image (Figures 3, 4), and subsequent cardiac MRI (Figure 5).

These examinations revealed a sizable mass measuring 5.0 x 7.0 x 6.3 x 4.0 cm within the right atrium. The mass appeared to be attached to the interatrial septum, protruding through the tricuspid valve (TV). Additionally, it was noted to be invading the posterior wall of the right atrium with involvement of the pericardium. No other lesions were observed, and a brain MRI returned negative findings.

Differential diagnosis for intracardiac mass was a cardiac tumor or thrombus. Considering she had a history of melanoma in the past, suspicions of cardiac melanoma were high. A multidisciplinary approach was taken, and cardiothoracic surgery, cardiology, and oncology teams were involved. The patient underwent debulking tumor surgery and was found to have a large lobular tumor mass occupying the entire right atrial cavity toward the TV inlet with severe obstruction. There was obstruction of flow from the IVC into the right atrium RA junction with partial protrusion of the mass into the IVC. There was an extensive tumor mass extending into the coronary sinus and distal coronary vein branches. The mass was removed piecemeal from the right atrium with extensive debulking of the mass, including from the coronary sinus. Frozen sections were suspicious of melanoma, which was confirmed in the final pathology report. A post-operative transesophageal echocardiogram showed moderate to severe tricuspid regurgitation. After a prolonged post-operative hospital stay complicated by bilateral deep venous thrombosis, pulmonary emboli requiring pulmonary embolectomy and vasopressor support, and right-sided pleural effusion requiring thoracentesis, the patient was stabilized and discharged.

Two weeks post-discharge, the patient followed up with an oncologist. In light of high tumor PDL-1 expression, the patient was started on immunotherapy. A few days later, the patient presented again to the emergency department with a complaint of blurry vision. CT head and brain MRI were performed, which demonstrated multiple lesions suggestive of metastatic disease. The patient underwent palliative whole-brain radiation. Unfortunately, the patient had a rapidly deteriorating course of the disease complicated by recurrence of the cardiac tumor occluding the right ventricle outflow tract. Despite chemotherapy and immunotherapy, the patient's condition continued to complicate, and she passed away after a sudden cardiac arrest.

## Discussion

Cardiac melanoma is a rare, aggressive tumor that is usually diagnosed at advanced stages. It is challenging to diagnose and in the early stages of spread, patients are asymptomatic. A comprehensive systemic review was performed by Alexander et al. in which thirty-three patients were identified [5]. The most common presenting symptom was shortness of breath and the physical exam finding was tachycardia at the time of diagnosis. Cardiac melanoma may also present as a cardiovascular complication such as arrhythmia, pericardial effusion, or heart failure [3].

The importance of multi-modality and advanced imaging is highlighted in a decision-making article by Borkovich et al. [6]. According to a study published in Dermatologic Therapy, in patients with metastatic melanoma, positron emission tomography and computed tomography (PET/CT) imaging could reduce the need for sentinel lymph node biopsy for staging. However, cost-effectiveness of PET/CT use was not studied [7]. Our patient also had multi-modality imaging including a CTA, an echocardiogram, and cardiac MRI along with a biopsy to establish the diagnosis. An echocardiogram is often used to provide preliminary information. In addition to the presence of mass, common echocardiogram findings are right ventricular outflow obstruction and valvular dysfunction (regurgitation, prolapse, or stenosis) [5]. PET/CT aids in identifying metastases, and CT and MRI scans are useful for characterization and tumor demarcation [8].

Treatment therapies involve surgical resection, chemotherapy, neoadjuvant chemotherapy with surgical resection, and immunotherapy [3,5]. Surgical management is often avoided due to advancement of the disease at the time of diagnosis. New therapeutic regimens (immunotherapy, BRAF, and MEK inhibitors) have shown relapse-free survival in patients with cutaneous melanoma but they are associated with cardiotoxicity [9]. The expected outcome of cardiac melanoma is poor with a median survival rate of 12 to 24 months [8].

There is limited data on surveillance imaging for cardiac melanoma in patients with a history of cutaneous metastasis melanoma. A review article by Long et al. highlights the need for further research on the utility of screening imaging for cardiac melanoma but there are no established guidelines [8]. 

This case highlights that acquisition of a complete history, routine follow-up, timely utilization of diagnostic imaging, and formulation and execution of targeted treatment strategies can help in effective management of this rare pathology.

## Conclusions

This case underscores the challenges associated with the diagnosis and management of rare cardiac malignancies such as cardiac melanoma. It emphasizes the importance of a collaborative, multidisciplinary approach, ongoing surveillance for metastatic disease, and the need for further research to improve outcomes in this challenging patient population.

Despite comprehensive efforts, the case highlights the aggressive nature of cardiac melanoma and the limitations of current treatment modalities in advanced stages of the disease.
